# Coping with dyspareunia, the importance of inter and intrapersonal context on women’s sexual distress: a population-based study

**DOI:** 10.1186/s12978-021-01206-8

**Published:** 2021-07-28

**Authors:** Ameneh Alizadeh, Farnaz Farnam

**Affiliations:** Department of Reproductive Health, Tehran University of Medical Sciences, Tehran, Iran

**Keywords:** Genito-pelvic pain/penetration disorder, Dyspareunia, Female sexual disorders, Sexual distress, Iran

## Abstract

**Background:**

Recently known as the genito-pelvic pain/penetration disorder (GPPPD), Dyspareunia is considered a negative factor affecting a couple’s sexual health. This paper analyzes pain in Dyspareunia cases and determines protective factors causing lower levels of sexual distress among patients.

**Methods:**

In a population-based cross-sectional study conducted in 2017, the cluster quota sampling technique was adopted to randomly select 590 Iranian married women aged 18–70 years from 30 health centers. The research tools included demographic data, a sexual distress scale, and Binik’s GPPPD questionnaire.

**Results:**

In this study, the prevalence of self-report Dyspareunia, confirmed moderate Dyspareunia, and confirmed severe Dyspareunia (based on Binik’s proposed criteria) were 33 %, 25.8 %, and 10.5 %, respectively. Interestingly, 32 (34 %) out of 94 women who experienced severe pain based on Binik’s criteria reported no sexual distress. Compared to women with distress, they also had more positive body images, higher self-confidence, higher levels of sexual satisfaction, and more intimacy in their relationships (P = 0.000). In contrast, 8.5 % of the participants reported significant sexual distress even without confirmed Dyspareunia.

**Conclusions:**

Improving intrapersonal characteristics such as self-confidence and body image as well as interpersonal factors such as sexual satisfaction and intimacy with a spouse can effectively treat Dyspareunia by alleviating sexual distress. The partner’s role in female pain and distress management would be more critical than previously thought.

## Plain English summary

Dyspareunia is a condition that can negatively affect sexual health among individuals and couples. This paper addresses the pattern of sexual pain as well as some interpersonal and intrapersonal factors that may lessen sexual pain by mitigating sexual distress. This population-based cross-sectional study was conducted on 590 randomly selected Iranian married women aged 18–70 years. According to the research results, the prevalence of self-report sexual pain was 33 %. However, based on strict Binik’s criteria and sexual distress, the prevalence of severe Dyspareunia decreased to 10.5 %. The most common location, time, and type of pain were reported to be in the vaginal entrance at the beginning of intercourse with irritation.

Interestingly, a substantial number of women reported no sexual distress, despite having severe Dyspareunia. Compared to patients with distress, they experienced higher levels of sexual satisfaction, more positive body images, higher levels of self-confidence, and more intimacy in their relationships (P = .000). In addition to the routine management of sexual pain, clinicians should consider these protective factors that can lead to better pain tolerance and less sexual distress among patients. The research findings emphasized the importance of attendance and collaboration of spouses in the treatment process.

At the same time, observing the sexual distress of women with no sexual dysfunctions would clarify that their own perception of sexual difficulty per se is an important factor. In other words, “sexual dysfunction” and “sexual problems” are both significant because defining sexual problems is subjective and depends on a woman and her partner’s values, desires, and sexual knowledge.

## Introduction

Sexual pain or Dyspareunia is a common problem that has significant effects on couples’ relationships. In DSM-5, the formerly separate Dyspareunia and vaginismus are merged and called the genito-pelvic pain/penetration disorder (GPPPD). The criteria for diagnosing the GPPPD include persistent or recurrent difficulties with at least one of the following conditions for at least six months resulting in significant distress: (a) vaginal penetration during intercourse; (b) marked vulvovaginal or pelvic pain during vaginal intercourse or penetration attempts; (c) marked fear or anxiety about vulvovaginal or pelvic pain in anticipation of, during, or as a result of vaginal penetration; and (d) marked tensing or tightening of the pelvic floor muscles during attempted vaginal penetration [1]. Since the bulk of previous studies used “sexual pain” and “dyspareunia” instead of the GPPPD, these terms were also used interchangeably in this study.

Sexual pain is a common problem. According to the findings of a study on women aged 40–80 years selected from 29 countries, the prevalence of sexual pain is nearly 21 % among Middle Eastern women [2]. Other studies report that 12 % of premenopausal women suffer from sexual pain [3]. Although cultural and religious factors can affect Dyspareunia experience and sexual distress, only a few standard studies have been conducted on the prevalence of sexual pain and associated factors in the Middle Eastern and Muslim Countries [4]. In a systematic review of Iranian studies in 2017, a wide range of Dyspareunia (9–95.9 %) was reported due to many methodological problems such as the lack of standard questionnaires, inappropriate sample size, and the lack of population-based surveys. The authors stated that more meticulous surveys would be needed to estimate the prevalence of Dyspareunia and its factors [5].

A small number of studies described the sexual pain pattern mostly as pain with irritation nature. The etiology of Dyspareunia is classified as acute genital infections, pelvic floor muscle dysfunction, and endometriosis, as well as psychological factors such as sexual abuse, trait anxiety, pain catastrophizing in addition to relationship factors such as negative and solicitous partner responses, insecure romantic attachments, and low sexual communication [6]. Several treatments have been proposed for Dyspareunia, such as vulvar hygiene and topical, oral, and injectable medications as well as surgery and cognitive-behavioral therapy. However, only a few randomized clinical trials (RCTs) have been conducted to verify the efficiency of different treatments, whereas most documents are based on clinical experience [6].

Sexual distress is crucial to the diagnosis of the GPPPD [1]. In fact, sexual distress means all the negative emotions such as anxiety, frustration, and feelings of inadequacy that people experience in their sexual relationships. These emotions can negatively affect overall well-being and quality of life [7]. Moreover, some sexual dysfunctions are not distressing for women; thus, it is essential to determine why and when sexual problems are distressing. In other words, are there any factors that may diminish sexual distress? [8]

Regarding the need for a survey based on the new concept of the GPPPD and the importance of conducting a standard study in society with different cultures from Western countries, our research team designed a population-based study within the 2017–2018 period. Although sexual pain can happen in women of all ages and rarely in men, this study focuses on married women. The results concerning the prevalence of the GPPPD, risk factors, and protective factors of the GPPPD are discussed explicitly in another paper [9]. The present paper addresses the characteristics of sexual pain, such as the location, time, and nature of pain. The paper also introduces the possible interpersonal and intrapersonal dimensions that may affect the sexual distress of women with Dyspareunia. In other words, the paper aims to indicate what factors may decrease sexual distress and help cope with Dyspareunia. These findings can be used by therapists and patients to design more effective interventions for the alleviation of pain.

## Methods

This population-based, cross-sectional study was conducted after the necessary scientific and ethic permits were obtained from Tehran University of Medical Sciences (IR.TUMS.FNM.REC.1396.2087; 2017.04.17).

### Sampling

In the metropolis of Tehran, with a population over eight million people, all health issues are taken care of by three medical universities. Tehran University of Medical Sciences is the oldest medical university in Iran, which covers the western districts and some parts of central and southern districts of Tehran with 30 main health centers and 73 sub-centers. All centers are included in an integrated health system registering the health information of all the residing families. The covered population in each center is nearly equal to those of other centers. Since a complete listing of all inhabitant women was available, the systematic sampling technique was employed. In this study, a two-stage random cluster sampling technique was also used in 30 main health centers. In the first stage, all 30 main health centers affiliated with Tehran University of Medical Sciences were included, whereas married women were selected equally at random from each center in the second stage. For the random selection of participants, a random starting point and a fixed sampling interval were used (by dividing the population size by the desired sample size). The individuals were then called by their phone numbers. They were invited to health centers to complete the written consent forms and questionnaires if they met the study criteria and the intention to participate in the survey. The sampling process was conducted by four trained midwives on the study area of 65 km^2^ for 20 weeks.

### Participants

Finally, 615 women were randomly selected from a list of 344,243 families who lived in the designated districts in 2017, and 590 participants completed the research questionnaires and entered the analysis. The statistical population was apprised of the survey purposes, and their verbal and written informed consent was obtained. The participants completed the questionnaires at the health centers, and a trained researcher attended to each participant. The inclusion criteria included being married, being aged 18–70 years, and cohabiting with a spouse for at least one year, whereas and the exclusion criterion was a report of drug abuse on the demographic questionnaire.

### Research tools

A checklist of 14 self-reported questions was employed to assess demographic characteristics as well as some interpersonal and intrapersonal factors. In this survey, the interpersonal factors included body image satisfaction and self-confidence, whereas intrapersonal factors consisted of intimacy and sexual and marital satisfaction, all of which were assessed by a single question. To differentiate between the self-reporting quality of pain or fear during intercourse with the confirmed GPPPD, the participants were asked, “Have you experienced considerable sexual pain during the last 6 months?” The presence of sexual distress (any anxiety or tension toward sexual pain) was confirmed by two questions from the Female Sexual Distress Scale-Revised (FSDS-R) [10]. In this study, Dyspareunia was defined based on DSM-5 and the integrated concepts of the GPPPD and then assessed based on Binik’s questionnaire [11].

The final diagnosis of Dyspareunia was confirmed when eight diagnostic questions of Binik’s scale and two questions of sexual distress both showed significant pain and distress. Those who reported pain or fear in single self-report questions were asked to answer the 11 complementary questions for the assessment of pain characteristics.

Binik’s guideline (2010) consisted of 19 questions in five dimensions: (A) percentage of success in vaginal penetration, (B) pain in vaginal penetration, (C) fear of vaginal penetration, (D) pelvic muscle dysfunction during vaginal penetration, and (E) medical comorbidity. For the fifth dimension, i.e., medical comorbidity, there was a list of medicaments, diseases, and surgeries that were more related to Dyspareunia (none of them showed any significant correlations in analysis). Eight questions concern the diagnosis of the GPPPD, whereas the other 11 questions pertain to the characteristics of pain in patients (the latter part was reported in this paper). Since this questionnaire is used in Iran for the first time ever, its face and content validities were determined. Ten participants checked the face validity. With more than 80 % of agreement between participants, all questions remained intact. It was the same for the content validity of the GPPPD confirmed by eight faculty members of Tehran University of Medical sciences. Two iterations confirmed the reliability with an interval of two weeks for 35 women and yielded a Cronbach’s alpha of 0.90 and internal consistency of 80 %.

### Statistical methods

A review of previous studies on Dyspareunia indicated that considering a prevalence rate of 26 % [12] could lead to an appropriate sample size estimation. Considering a two-sided confidence interval of 95 %, with a width equal to 0.08 (margin of error = 0.04), a design effect of 1.2, and a non-responding rate of 10 %, 615 women were selected as the research sample. The values of distribution, mean, and standard deviation were then used for data analysis. Chi-square and independent t-test were adopted to determine the homogeneity of the two groups. The linear logistic regression analysis was conducted to estimate correlations between sexual distress and associated factors. The collected data were then analyzed in SPSS 22 (SPSS Inc., Chicago, IL, USA).

## Results

The demographic characteristics of 590 individuals showed that the mean of women’s age and the duration of marriage were 35.5 and 13.8 years, respectively. The majority of women had two children (43.5 %), appropriate financial status (66.8 %), and high school diplomas (75 %). Approximately 90 % of women were housewives, whereas 32 % reported that they were nervous during sexual activities due to the lack of privacy. Only 4.2 % had vulvar pain during vestibular touch, and 36 women (6.1 %) were menopausal. Most women reported moderate self-confidence (46 %) and body image satisfaction (45 %). Nearly 70 % had sufficient intimacy with their husbands, and most of them (86 %) reported moderate-to-high levels of sexual satisfaction. Moderate and high levels of marital satisfaction were reported by 27 and 61 % of participants, respectively (Table 1).

**Table 1:** Demographic characteristics

	Total number = 590 Number (%)
*Women’s age (year***)**	
30 <	146 (24.7)
30–39	283 (48)
40–49	124 (21)
<50	37 (6.3)
Min-Max	18–61
Mean (SD)	8.1 ± 35.5
*Duration of marriage (year)*	
1–9	237 (40.2)
10–19	216 (36.6)
20–29	96 (16.3)
<30	41 (7.0)
Min–Max	1–47
Mean (SD)	9.1 ± 13.7
*Nnumber of children*	
0	37 (6.3)
1	183 (31.0)
2	255 (43.2)
3	98 (16.6)
<4	17 (2.9)
*Financial situation*	
Appropriate	402 (68.1)
In middle range	164 (27.8)
Inappropriate	24 (4.1)
*Women’s education*	
Primary/secondary school	148 (25.1)
High school	288 (48.8)
Under graduate	136 (23.1)
Ms. and PhD	18 (3.1)
*Women occupation*	
Household	527 (89.3)
Governmental job (Office jobs)	36 (6.1)
Non-governmental job	27 (4.5)
*Having privacy room*	
Yes	467 (79.3)
No	122 (20.7)
*Having privacy room*	
Yes	467 (79.3)
No	122 (20.7)
*Vestibular pain*	
Yes	25 (4.2)
No	564 (95.6)
*Menopause*	
Yes	36 (6.1)
No	554 (93.9)
*Self-confidence*	
High	197 (33.5)
Moderate	273 (46.4)
Low	118 (20.1)
*Body image satisfaction*	
High	195 (33.4)
Moderate	265 (44.9)
Low	128 (21.7)
*Intimacy with spouse*	
High	404 ( 68.5)
Moderate	116 (19.6)
Low	70 (11.9)
*Sexual Satisfaction*	
High	353 (59.8)
Moderate	154 (26.1)
Low	83 (14.1)
*Marital satisfaction*	
High	365 (61.8)
Moderate	158 (26.8)
Low	67 (11.4)

As discussed in our previous paper [9] and based on Binik’s criteria, the final prevalence rates of severe and moderate Dyspareunia were 10.5 and 25.8 %, respectively. However, 33 % of participants reported experience of sexual pain in the single self-report question.

Table 2 demonstrates the sexual distress among participants. Moreover, out of 84 % of participants who were either healthy without pain or those with self-reported Dyspareunia (the pain was not confirmed based on Binik’s criteria), 8.5 % reported a considerable level of sexual distress. In contrast, out of 16 % of women whose Dyspareunia was confirmed based on strict Binik’s criteria, 5.4 % reported no sexual distress despite severe pain (Table 2).

**Table 2:** Sexual distress among participants

	Total participants N (%)	With sexual distress N (%)	Without sexual distress N (%)
Without pain or those with self-reported Dyspareunia (not confirmed)	496 (84)	50 (8.5)	446 (75.5 )
Dyspareunia confirmed only based on Binik criteria	94 (16)	62 (10.5)	32 (5.5)
Dyspareunia confirmed based on both Binik criteria and sexual distress		62 (10.5)*	
Total	590 (100)	112 (19)	478 (81)

*Confirmed severe case of GPPPD: 10.6 %

Table 3 depicts the status of eight diagnostic questions for all participants. Even though the pain level was severe in all 62 participants suffering from the GPPPD, 100 % of them stated that they had experienced more than ten intercourses within the past six months (vs. 82 % among the otherwise healthy women). Significant pain and fear during intercourse were reported in 53.2 and 37.1 % of the women suffering from the GPPPD (vs. 3.6 and 1.3 % among the non-sufferers of the GPPPD). Furthermore, 25.8 and 33.9 % of the women suffering from the GPPPD reported severe distress during intercourse and stiffness of vagina muscles (vs. 2.8 and 3.8 % among the otherwise healthy women) (Table 3).

**Table 3:** Diagnostic questions in all participants (8 questions of BINIK scale for the diagnosis of GPPPD)

	Dyspareunia Confirmed (N = 62) NO (%)	Healthy women* (N = 528) NO (%)	Total sample (N = 590) NO (%)
*Number of intercourse in the last six months*			
Less than 10 times	0 (0.0)	95 (18)	95 (16.1)
More than 10 times	62 (100)	433 (82)	495 (83.9)
*Number of full penetration in the last six months*			
Less than 50 % of attempt	2 (3.2)	25 (4.7)	27 (4.6)
More than 50 % of attempt	60 (96.8)	503 (95.3)	563 (95.4)
*Feel of pain during intercourse*			
No pain	2 (3.2)	207 (39.2)	209 (35.4)
A little pain	13 (21.0)	173 (32.8)	186 (31.5)
Some pain	14 (22.6)	129 (24.4)	143 (24.2)
Moderate pain	22 (35.5)	17 (3.2)	39 (6.6)
Quite a bit of pain	11 (17.7)	2 (0.4)	13 (2.2)
*Anxious about or fear of pain during intercourse*			
Not at all	6 (9.7)	329 (63.3)	335 (56.8)
A little	11 (17.7)	135(25.6)	146 (24.7)
Somewhat	22 (35.5)	57 (10.8)	79 (13.4)
Moderately	16 (25.8)	5 (0.9)	21 (3.6)
Quite a bit or always	7 (11.3)	2 (0.4)	9 (1.5)
*Anxious about other things during intercourse*			
Not at all	10 (16.1)	331 (62.7)	341 (57.8)
A little	18 (29)	123 (23.3)	141 (23.9)
Somewhat	13 (21)	64 (12.1)	77 (13.1)
Moderately	15 (24.2)	6 (1.1)	21 (3.6)
Quite a bit or always	6 (9.7)	4 (0.8)	10 (1.7)
*General tense during intercourse*			
Not at all	10 (16.1)	303 (57.4)	313 (53.1)
A little	16 (25.8)	141 (26.7)	157 (26.6)
Somewhat	20 (32.3)	69 (13.1)	89 (15.1)
Moderately	11 (17.7)	8 (1.5)	19 (3.2)
Quite a bit or always	5 (8.1)	7 (1.3)	12 (2.0)
*Vaginal muscles tighten up during intercourse*			
Not at all	8 (12.9)	259 (49.1)	267 (45.3)
A little	13 (21.0)	171 (32.4)	184 (31.2)
Somewhat	20 (32.3)	78 (14.8)	98 (16.6)
Moderately	15 (24.2)	16 (3.0)	31 (5.3)
Quite a bit or always	6 (9.7)	4 (0.8)	10 (1.7)
*Interfere muscle tension with ability to intercourse*			
Not at all	9 (14.5)	291 (55.1)	300 (50.8)
A little	15 (24.2)	148 (28.0)	163 (27.6)
Somewhat	16 (25.8)	76 (14.4)	92 (15.6)
Moderately	17 (27.4)	10 (1.9)	27 (4.6)
Quite a bit or always	5 (8.1)	3 (0.6)	8 (1.4)

*Without pain or self-report pain that their pain did not confirm based on both Binik criteria and sexual distress

Table 4 shows sexual pain characteristics in response to 11 complementary Binik’s questions answered by only 196 participants (33 % of the sample) with self-reports of Dyspareunia. In addition, these questions were quite personal and not of any diagnostic nature; thus, the participants did not have to answer them. The number of individuals who had answered these questions was not equal for each question in Table 4.

**Table 4:** Complementary questions in women with self- report pain (11 question for sexual pain description)

Sexual pain characteristics	Self-report of Dyspareunia (N = 196) NO (%)	Dyspareunia Confirmed* (N = 53) NO (%)	Dyspareunia Not confirmed (N = 143) NO (%)	p-value¥
*The most important reason for intercourse*				0.430
To get pregnant	5 (2.6)	1 (1.9)	4 (2.8)	
To please partner	52 (26.5)	14 (26.4)	38 (26.6)	
To have pleasure	25 (12.8)	6 (11.3)	19 (13.3)	
To improve couple relationship	104 (53.1)	29 (54.7)	75 (52.4)	
To improve sexual self-esteem	5 (2.6)	0 (0.0)	5 (3.5)	
Other	5 (2.6)	3 (5.7)	2 (1.4)	
*Time of pain*				0.243
Before intercourse	7 (3.6)	4 (7.5)	3 (2.1)	
At the beginning of intercourse	71 (36.2)	21 (39.6)	50 (35.0)	
During thrusting	68 (34.7)	21 (39.6)	47 (32.9)	
During orgasm	3 (1.5)	1 (1.9)	2(1.4)	
After intercourse	14 (7.1)	3 (5.7)	11 (7.7)	
During gynecological examinations	13 (6.6)	2 (3.8)	11 (7.7)	
While wearing tight pants	3 (1.5)	1 (1.9)	2 (1.4)	
While exercising	0 (0.0)	0 (0.0)	0 (0.0)	
Not related to intercourse	11 (5.6)	0 (0.0)	11 (5.6)	
Other	5 (2.6)	0 (0.0)	5 (3.5)	
I don’t know	1 (0.5)	0 (0.0)	1 (0.7)	
*Location of pain*	193 (100.0)	27 (100.0)	166 (100.0)	0.058
I don’t know	9 (4.7)	2 (3.8)	8 (5.7)	
Clitoris	0 (0.0)	0 (0.0)	0 (0.0)	
Labia minor	0 (0.0)	0 (0.0)	0 (0.0)	
Labia major	5 (2.6)	4 (7.5)	1 (0.7)	
Vaginal opening	96 (49.7)	24 (45.3)	72 (51.1)	
Urethral opening	0 (0.0)	0 (0.0)	0 (0.0)	
Vestibule	2 (1.0)	0 (0.0)	2 (1.4)	
Uterus	35 (18.1 )	9 (17.0)	26 (18.4)	
Cervix	25 (13.0)	10 (18.9)	15 (10.6)	
Ovary	15 (7.8)	4 (7.5)	11 (7.8)	
Fallopian tubes	6 (3.1)	0 (0.0)	6 (4.3)	
*Description of quality of pain*	195 (100.0)	52(100.0)	143 (100.0)	0.613
Throbbing,	19 (9.7)	4 (7.7)	15 (10.5)	
Shooting	14 (7.2)	6 (11.5)	8 (5.6)	
Cramping	51 (26.2)	9 (17.3)	42 (29.4)	
Gnawing	2 (1.0)	1 (1.9)	1 (0.7)	
Hot-burning	86 (44.1)	25 (48.1)	61 (42.7)	
Heavy	10 (5.1)	3 (5.8)	7 (4.9)	
tiring-exhausting	9 (4.6)	3 (5.8)	6 (4.2)	
fearful	4 (2.1)	1 (1.9)	3 (2.1)	
*Effects of pain on the ability to intercourse*				0.004**
Not at all	60 (30.6)	10 (18.9)	50 (35.0)	
A little	73 (37.2)	15 (28.3)	58 (40.6)	
Somewhat	49 (25.0)	23 (43.4)	26 (18.2)	
Moderately	11 (5.6)	4 (7.5)	7 (4.9)	
Quite a bit or always	3 (1.5)	1 (1.9)	2 (1.4)	
*Effects of pain on desire*				0.038*
Not at all	70 (35.7)	14 (26.4)	56 (369.2)	
A little	75 (38.3)	17 (32.1)	58 (40.6)	
Somewhat	39 (19.9)	16 (30.2)	23 (16.1)	
Moderately	9 (4.6)	4 (7.5)	5 (3.5)	
Quite a bit or always	3 (1.5)	2 (3.8)	1(0.7)	
*Effects of fear on the ability to intercourse*				0.036*
Not at all	75 (38.3)	13 (24.5)	62 (43.4)	
A little	64 (32.7)	16 (30.2)	48 (33.6)	
Somewhat	41 (20.9)	17 (32.1)	24 (16.8)	
Moderately	9 (4.6)	4 (7.5)	5 (3.5)	
Quite a bit or always	7 (3.6)	3 (5.7)	4 (2.8)	
*Medical/surgery conditions that might have caused difficulties*				0.769
No	168 (85.7)	47(88.7)	121 (84.6)	
Yes	19 (9.7)	4 (7.5)	15 (10.5)	
I don’t know	9 (4.6)	2 (3.8)	7 (4.9)	
*Recent gynecological examination for routine checkup*				0.451
No	106 (54.1)	31 (58.5)	75 (52.4)	
yes	90 (45.9)	22 (41.5)	68 (47.6)	
*Tell the health provider about sexual pain*				0.654
No	132 (67.3)	37(69.8)	95 (66.4)	
Yes	64 (32.7)	16 (30.2)	48 (33.6)	
*The physical reason for your pain*				0.226
No	74 (37.8)	18 (34.0)	56 (39.2)	
Yes	33 (16.8)	6 (11.3)	27 (18.9)	
I don’t know	89 (45.4)	29 (54.7)	60 (42.0)	

* Dyspareunia confirm based on both Binik criteria and sexual distress, ¥ tested between the last two columns (confirmed and not confirmed dyspareunia), *P < .05, **P < .01

Regarding most of the participants (53.1 %), the main reason for sexual intercourse was to improve the marital relationship. Most women (36.2 %) experienced the pain at the beginning of the intercourse, whereas the majority of them (49.7 %) reported the pain in the vagina entrance. Most of the women (44.1 %) described the pain as a sense of irritation or heat. The negative impact of sexual pain on the desire for sexual intercourse and the ability to have sexual intercourse were significantly different in the two groups (P = .038 and P = .004, respectively). Abstinence from sexual intercourse was significantly higher among the women whose Dyspareunia was confirmed by the standard criteria than among the self-reported cases. Moreover, the negative impact of fear of sexual intercourse on the ability to have sexual intercourse was significantly different in the two groups (P = .036), whereas 67.3 % of the population who felt pain never discussed their problems with the treatment team (Table 4).

## Discussion

This paper discussed the characteristics of sexual pain and determined that protective factors would cause less sexual distress among the patients. Accordingly, some women with severe Dyspareunia complained nothing about any sexual distress, whereas some women without any sexual pain diagnosis experienced significant sexual distress.

### Sexual distress, despite lack of Dyspareunia confirmation

In this survey, 19 % of the population reported significant distress during sexual intercourse, although the sexual pain was not confirmed and interpreted carefully in 8.5 % of cases. This group either experienced no pain or reported pain that was not confirmed based on the standard pain criteria. The existence of sexual distress, despite lack of significant sexual pain, can have several reasons. First, considering various criteria may lead to an underestimation of sexual pain. This study was conducted in accordance with the new DSM-5 standards as well as the criteria recommended by Binik’s questionnaire. Moreover, only the presence of considerable and severe levels of pain (Options 3 and 4) were deemed the diagnostic criteria. Therefore, those individuals who experienced medium or mild pain were excluded from the Dyspareunia diagnosis. It should be noted that pain leaves a considerable effect on the quality of life. In addition, people interpret pain differently. Even those suffering from slight pain might experience significant distress in their sexual lives due to the nature of pain or pain catastrophizing. Various studies suggest that an influential factor in sexual pain or fear of intercourse might be pain catastrophizing [4]. The second interpretation of sexual distress in the non-sufferers of Dyspareunia can be related to other sexual and non-sexual problems such as sexual trauma, mental health issues, partner’s sexual violence, and the obligation to have sexual relationships are not dealt with in this study. The sexual issues are broadly related to other aspects of life. This finding shows that the assessment of sexual disorders would not be enough for the evaluation of sexual health. In many cases, sexual distress emerges due to certain causes other than sexual dysfunction. This result reconfirms Bancroft’s views showing that the physical aspects of female sexual responses, including arousal and orgasm, are the poor predictors of female distress [13]. According to King et al., a woman’s own perception that she has sexual difficulty is per se an important factor because defining a sexual problem is subjective and depends on her personal values, wishes, and sexual knowledge and those of her partner’s [14]. Therefore, comprehensive medical history is essential to appropriate interventions and the discovery of reasons behind sexual concerns and distress. Sexual distress may emerge for various reasons, including lack of trust in or love for one’s spouse, concerns about pregnancy, and the presence of children [15].

### Lack of sexual distress, despite Dyspareunia confirmation

Surprisingly, a considerable number of women reported no sexual distress, despite experiencing severe levels of confirmed Dyspareunia. Consequently, they were excluded from the final diagnosis of the GPPPD. However, this finding is significant because it proposes that some variables protect those participants from the experience of distress or conflict in their sexual relationships. In other words, their coping strategies could be considered valuable solutions for other sufferers. According to the analysis results, some interpersonal variables such as self-confidence (P = .000) and positive body image (P = .000) as well as some intrapersonal factors such as sufficient intimacy (P = .000) and sexual satisfaction (P = .000) were significantly higher in this subgroup than among those suffering from distress in addition to pain.

Self-confidence and positive body image had direct and indirect roles in increasing sexual satisfaction and declining distress. Even though the previous studies showed that sexual pain might affect self-confidence and body image [16], this study took one step further and indicated that those factors might even be of some protective effect against sexual distress.

The protective effect of sexual satisfaction in these women can be interpreted by replacing and enjoying other sexual activities instead of penetration and consequently less attention to and anxiety about Dyspareunia. Many studies have described the role of intimacy in better adjustment with sexual pain. Intimacy can help with better communication and selection of alternative sexual activities. Leeners et al. and Stephenson et al. indicated that the increased emotional intimacy decreased both sexual pain and anxiety in the afflicted individuals [8, 17]. Benoit-Piau et al. also reported that a partner’s supports could moderate pain catastrophizing in women with vulvodynia [18]. According to Bancroft, the best predictor of women’s sexual distress was to have emotional relationships with their spouses [13]. Apparently, a spouse’s role is very critical in the management of sexual pain and distress, whereas spousal attendance is essential to the treatment process. The present study proposes some strategies for overcoming severe pain. These strategies can benefit both patients and therapists. However, other surveys need to determine the other factors that may result in the better toleration of Dyspareunia.

### Characteristics of Dyspareunia

This study analyzed some characteristics of sexual pain among Iranian women who suffered from Dyspareunia. According to Table 3, all the individuals whose final diagnosis had been confirmed had more than ten sexual intercourses during the previous six months. A review of Table 4 and the reasons for having sexual intercourse showed that 81 % of the suffering individuals interpreted spousal satisfaction and betterment of marital relations as the main reasons for having sexual intercourse. Despite severe pain, sexual intercourse can be devastating. If therapists fail to alleviate the pain, they must help couples enjoy sexual activities other than penetration. Painful intercourse can disgust people not only of the penetration but also of any other sexual activities. Hence, spousal accompaniment during the treatment process is beneficial to the resolution of the pain issue and the replacement of vaginal penetration with other less painful sexual activities [19]. In response to the location and time of pain, most participants experienced pain in the vaginal opening at the beginning of intercourse. These findings help the therapists pay more attention to specific reasons for this type of sexual pain, such as infections and the decreased lubricity of the vagina due to insufficient stimulation.

The individuals with and without Dyspareunia showed a significant difference in the effect of pain on the ability to have intercourse, the effect of pain on sexual desire, and the effect of fear on the ability to have intercourse. These findings confirm the diagnostic criteria and show that a diagnosis of sexual disorder is valid only when pain and fear leave a significant effect on the ability to have intercourse and sexual desire. Various studies show that pain disorder decreases sexual desire by creating fear and a vicious circle [20] that can lead to abstinence from sexual intercourse [21].

This study employed a single question and a self-reporting mechanism to analyze the factors related to sexual pain. However, these factors were not assessed by the standard questionnaire. The advantages of this study include a large statistical population, the randomized sample selection, the population-based nature of research, the use of a standard questionnaire for diagnosis, and employment of the new DSM-5 definitions.

## Conclusions

Even though pain concurs with the decreased quality of life and various side effects, sexual pain has adverse, significant effects on individuals and couples by reducing sexual desire, decreasing sexual intercourse, and declining sexual satisfaction. Nevertheless, most women (67 %) do not inform health providers of their sexual pain or fear and continue to have intercourse due to fear of losing their spouses. The findings of this study showed that improving self-confidence, having a positive body image, enhancing sexual satisfaction, and increasing marital intimacy in addition to the routine management of sexual pain or fear of intercourse were the protective factors that could cause better toleration of pain and less sexual distress. Apart from medical treatments, working on patients’ characteristics can also help them cope with the sexual pain problem. It is also important to emphasize a partner’s role in alleviating a woman’s sexual pain with appropriate support and communication in order to point out the importance of considering a partner’s role in treating Dyspareunia.

In this survey, 19 % of participants reported significant sexual distress (10.5 % of confirmed Dyspareunia and 8.5 % of unconfirmed Dyspareunia cases). It is crucial to pay attention to women’s concerns about sexuality in unconfirmed Dyspareunia cases. In fact, the causes of sexual distress are not merely related to sexual dysfunctions in many cases, and it is necessary to obtain comprehensive medical history to find the causes of sexual concerns and distress.

## Data Availability

The data are available from the corresponding author on reasonable request.

## Abbreviations

GPPPD: Genito-pelvic pain/penetration disorder
FSDS-R: Female sexual distress scale-revised
